# Dimensional Accuracy Evaluation of Single-Layer Prints in Direct Ink Writing Based on Machine Vision

**DOI:** 10.3390/s25082543

**Published:** 2025-04-17

**Authors:** Yongqiang Tu, Haoran Zhang, Hu Chen, Baohua Bao, Canmi Fang, Hao Wu, Xinkai Chen, Alaa Hassan, Hakim Boudaoud

**Affiliations:** 1 College of Marine Equipment and Mechanical Engineering, Jimei University, Xiamen 361021, China; tuyq@jmu.edu.cn (Y.T.); 202412855070@jmu.edu.cn (H.Z.); 202321365108@jmu.edu.cn (B.B.); 202321361004@jmu.edu.cn (C.F.); 202321361131@jmu.edu.cn (H.W.); 202321361104@jmu.edu.cn (X.C.); 2 Innovation Process Research Institute, University of Lorraine, F-54000 Nancy, France; alaa.hassan@univ-lorraine.fr (A.H.); hakim.boudaoud@univ-lorraine.fr (H.B.)

**Keywords:** machine vision, dimensional accuracy evaluation, single layer, direct ink writing

## Abstract

The absence of standardized evaluation methodologies for single-layer dimensional accuracy significantly hinders the broader implementation of direct ink writing (DIW) technology. Addressing the critical need for precision non-contact assessment in DIW fabrication, this study develops a novel machine vision-based framework for dimensional accuracy evaluation. The methodology encompasses three key phases: (1) establishment of an optimized hardware configuration with integrated image processing algorithms; (2) comprehensive investigation of camera calibration protocols, advanced image preprocessing techniques, and high-precision contour extraction methods; and (3) development of an iterative closest point (ICP) algorithm-enhanced evaluation system. The experimental results demonstrate that our machine vision system achieves 0.04 mm × 0.04 mm spatial resolution with the ICP convergence threshold optimized to 0.001 mm. The proposed method shows an 80% improvement in measurement accuracy (0.001 mm) compared to conventional approaches. Process parameter optimization experiments validated the system’s effectiveness, showing at least 76.3% enhancement in printed layer dimensional accuracy. This non-contact evaluation solution establishes a robust framework for quantitative quality control in DIW applications, providing critical insights for process optimization and standardization efforts in additive manufacturing.

## 1. Introduction

Additive manufacturing (AM) technology is an advanced manufacturing technology based on computer-aided design (CAD) three-dimensional (3D) models, which utilizes the principle of layer-by-layer stacking to achieve the formation of complex components. It overcomes the problems of high cost, long cycle, and even inability to manufacture traditional manufacturing technology in the production of complex structural or functional components. It is an important means of modern advanced manufacturing technology and an important supplementary means to traditional manufacturing technology in Industry 4.0 [1,2,3].

Direct ink writing (DIW), also known as direct write fabrication [4] or robocasting [5], was first proposed by Cesarano et al. from Sandia National Laboratory in the United States in 1998 [6]. DIW is one of the AM technologies that belongs to the material extrusion technology category in AM according to its working principle [7]. The working principle of DIW is as follows. First, ink is filled into a needle tube with a precision nozzle. Then, the ink is extruded from the nozzle into continuous extruded filaments, and the nozzle’s movement path is controlled by a computer to deposit the extruded filaments into two-dimensional shapes for each layer. Finally, after completing the deposition of one layer, the nozzle is vertically moved a distance relative to the previous layer, and a 3D solid part is manufactured through the principle of layer by layer stacking [8].

After the disclosure of DIW, many researchers realized that DIW has obvious advantages over other AM technologies in terms of material range, cost, efficiency, etc. And much research has been conducted on material preparation, process improvement, and application promotion of DIW. At present, DIW has been successfully applied in fields such as tissue engineering [9], batteries [10], electronic circuits [11], wearable sensors [12], 4D printing [13], soft robots [14], and radio frequency devices [15], and is a hot topic in AM research. With the development of materials and manufacturing science, it can be foreseen that more and more materials will be studied and prepared into inks suitable for DIW, and formed into 3D parts, achieving applications in more fields [16].

Similarly to other AM technologies, the quality indicators for evaluating DIW can include molding quality, molding efficiency, process stability, mechanical performance quality, and functional quality [17,18,19]. Among them, single-layer dimensional accuracy belongs to the molding quality, which is the most basic quality indicator and has a direct impact on other quality indicators for DIW. It is also the most concerned quality indicator in current research on DIW [20,21]. However, there is currently limited research on the dimensional accuracy of printed single layer for DIW.

Due to the fact that the formed elements are in a highly viscous and elastic fluid state that is easily destroyed before solidification during the DIW process, traditional contact based dimensional measurement will damage the DIW elements. Therefore, the demand for dimensional accuracy evaluation of printed single layer in DIW is non-contact measurement. Machine vision-based dimensional accuracy evaluation method has the advantages of high accuracy and non-contact, and has been applied by relevant researchers in the dimensional accuracy evaluation of extrusion AM. Xu et al. [22] used a structured light scanner installed on a robotic arm to measure the entire surface of parts in cement ink based DIW and converted the measurement data into a 3D point cloud. Finally, the geometric dimension tolerance method was used to analyze the deviation between the 3D point cloud and the designed 3D model to evaluate the dimensional accuracy. Wi et al. [23] developed a 3D structured light scanning system for extrusion 3D printing of clay materials, using a projector and a camera. The system scanned and printed parts from four different directions to obtain 3D graphics and generated 2D graphics along the height and edges of the 3D graphics. The dimensional parameters of the printed and designed parts, including geometric shape, layer height, thickness, distortion angle, and area, obtained from the 2D graphics were compared to quantitatively evaluate the dimensional accuracy. Petsiuk et al. [24] focused on melt extrusion 3D printing using a monocular camera to monitor the external shape and single-layer internal structure of the printed parts in real time and achieved the evaluation of global and local parameters of the printed parts. Shen et al. [25] introduced a multi-perspective omnidirectional visual inspection method, which identified printing defects in real-time on the surface of printed parts during the inspection process.

Therefore, non-contact measurement based on machine vision is currently the best choice for evaluating the dimensional accuracy of printed single layer in DIW. However, at present, there is a lack of mature single-layer dimensional accuracy evaluation methods for DIW, which limits the application of DIW. This study focuses on the demand for accurate and non-contact evaluation of single-layer dimensional accuracy in DIW and conducts research on machine vision based dimensional evaluation of single layer in DIW. Specifically, the main contributions of this article are as follows.

(1) A quantitative single-layer dimensional accuracy evaluation method for DIW is provided using a novel single-camera based machine vision system;
(2) An image processing algorithm flow of the quantitative single-layer dimensional accuracy evaluation method is proposed;
(3) Iterative closest point (ICP) algorithm is used in the method to improve the evaluation accuracy.

The remainder of this paper is structured as follows. Section 2 reviews related works and existing methodologies in machine vision based non-contact measurement systems for the dimensional accuracy evaluation of different product types. Section 3 details the hardware setup and image processing algorithms, including camera calibration, image preprocessing and contour extraction, and quantitative acquisition of single-layer dimensional accuracy. Section 4 discusses the results, comparing our approach with existing techniques. Finally, Section 5 concludes the paper, summarizing the key findings and potential future research directions.

## 2. Related Works

With advances in high-resolution camera and image processing technologies, numerous machine vision based non-contact measurement systems and methods have been proposed for the dimensional accuracy evaluation of different product types. Sun et al. [26] developed a machine vision method for high precision 2D measurement by improving the calibration model. The lens distortion was corrected on the pixel plane before measuring, and accurate magnification factors of imaging system could be obtained. The experimental results indicated that the proposed method possessed a precision of 0.005 mm for measuring a shaft diameter of about 40 mm. Li et al. [27] used a CCD camera to collect the parts’ shapes in real time, and a series of preprocessing operations, such as image grayscale processing and wavelet denoising, were used to improve accuracy. The experimental results showed that the repetitive error of the measuring system was less than 0.01 mm. Dang et al. [28] introduced a method for reconstructing the 2D profiles of large-size ring-shaped objects using a simple single-camera system by capturing the inspection object with multiple partial images. The proposed system enhanced the accuracy of the measurement results by increasing the resolution of the image up to 3.6 times. The system could accurately measure the complex profile of a 192 mm diameter ring-shaped object with an error under 0.063 mm. Tan et al. [29] studied the measurement of shaft diameter with the structured light system composed of a laser linear light source and a camera. The test results showed that the measurement model of shaft diameter is correct as the maximum average measurement error is 0.019 mm. Nogueira et al. [30] proposed a method for automatically measuring specifications on the external contours of mechanical parts using a monocular machine vision system by image processing. This prototype was validated via both traceability and comparative tests, demonstrating its ability to perform measurements quickly and accurately in either straight or circular sections. When making planar measurements of mechanical parts, the accuracy was 0.008 mm.

Table 1 summarizes the main related works, detailing their measured product types, used techniques, and accuracy results. Collectively, these studies used single-camera system-based machine vision with image processing to achieve high measurement accuracy.

## 3. Materials and Methods

### 3.1. Hardware Setup

**Figure 1:**
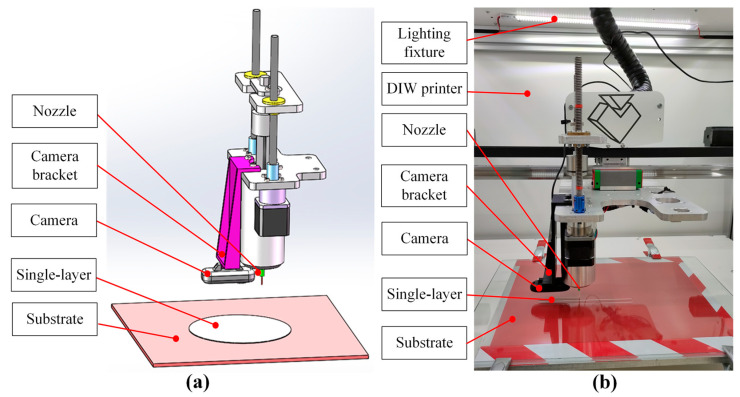
Camera installation diagram for hardware construction: (**a**) installation design diagram; (**b**) physical picture.

**Figure 2:**
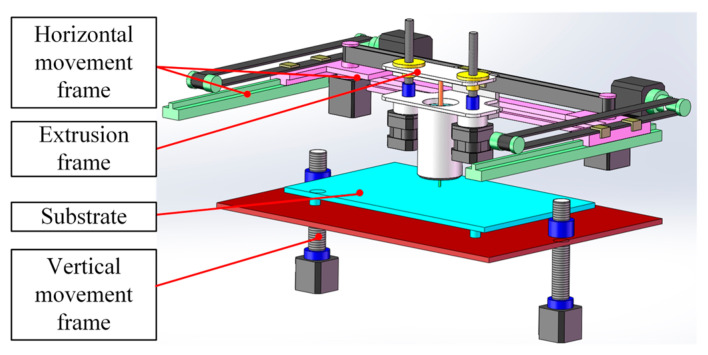
Illustration of the main components for implementing extrusion 3D printing function in DIW 3D printer.

In order to obtain accurate and easy to process printed single-layer images, the key principle in hardware construction is to ensure that the camera light path is perpendicular to the printed single layer. As shown in Figure 1, first, a camera bracket was designed. The bracket’s mounting surface was aligned parallel to the camera’s installation axis, which itself formed a 90-degree angle with the optical path. This configuration guaranteed proper orthogonality between the imaging system’s light trajectory and the substrate. The bracket was installed on the extrusion mechanism, and the camera was installed on the bracket. To enhance image clarity, we first applied a pure red base layer beneath the glass substrate. This red backing creates strong visual contrast with the white ink patterns, making the printed elements’ edges distinctly visible. The improved color differentiation specifically aids computerized edge detection during image analysis. At the same time, a lighting fixture was installed on the top of the inside of the DIW printer to prevent the light from being too dim and resulting in unclear single-layer printed images. In this study, the ink was a commercially available microcrystalline wax (MW)-based ink, Nivea Crème Art. No. 80,104 (Beiersdorf Global AG, Hamburg, Germany), which was selected as the ink reference, as it was the printability reference and the representative for ink preparation and process parameter selection for DIW. The DIW printer was a piston-driven DIW 3D printer TM-081 (Tobeca Company, Nancy, France). As shown in Figure 2, the main components for implementing extrusion 3D printing function in the DIW 3D printer are a horizontal movement frame, a vertical movement frame, and an extrusion frame. The horizontal movement frame can move the extrusion frame in a horizontal plane. The vertical movement frame can move the substrate relative to the extrusion frame in the vertical direction.

**Figure 3:**
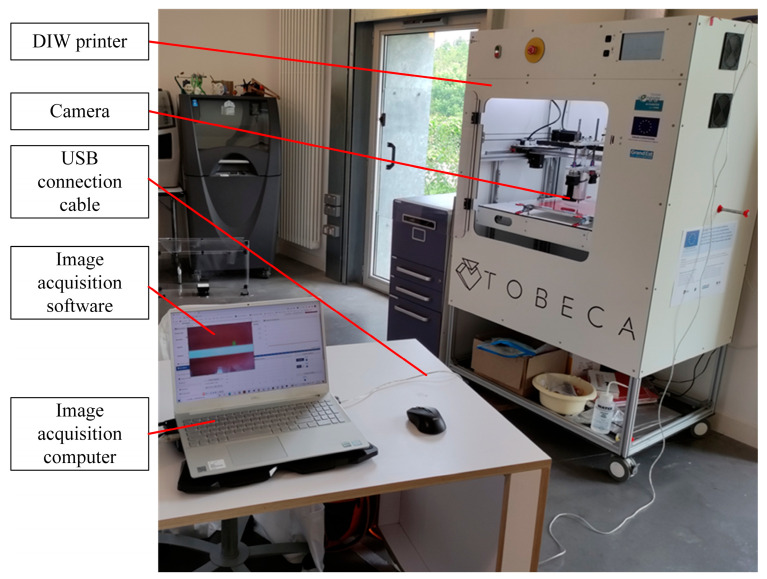
Overall hardware architecture diagram.

As shown in Figure 3, the printed single-layer image obtained by the camera was connected to the image acquisition computer through a USB cable. The image acquisition computer was equipped with an image acquisition software written using the open-source machine vision software OpenCV 4.5.0. Based on the hardware constructed as shown in Figure 1, Figure 2 and Figure 3, the process of obtaining single-layer images for DIW was as follows: after the single-layer printing was completed, the horizontal movement frame of the DIW printer controlled the extrusion mechanism to move to a position above the midpoint of the printed layer so that the camera light path was at the center of the outer contour. Then, the vertical movement frame of the DIW printer controlled the substrate to move downward relative to the extrusion mechanism so that the printed single-layer image was included in the camera’s field of view. The camera model was Logitech Brio 501 (Logitech, Lausanne, Switzerland), and the optical sensor was a Charge Coupled Device (CCD) chip with a maximum resolution of 1280 × 1080 pixels. The measurement accuracy based on machine vision depended on the field of view size and maximum resolution. The maximum outer contour of a printed single layer was 50 mm × 50 mm. According to Equation (1) [31], the measurement accuracy of the system was calculated to be about 0.04 mm × 0.04 mm, which was on the same level as the repeatability accuracy of DIW (0.05 mm) and met the measurement accuracy requirements of DIW.

(1) $$\varepsilon_{v}=\frac{S_{L\times W}}{P_{x\times y}},$$

where $\varepsilon_{v}$ is the measurement accuracy of the machine vision-based system, $S_{L\times W}$ is the field of view size, and $P_{x\times y}$ is the number of pixels obtained from the image.

### 3.2. Design of Image Processing Algorithm Flow

**Figure 4:**
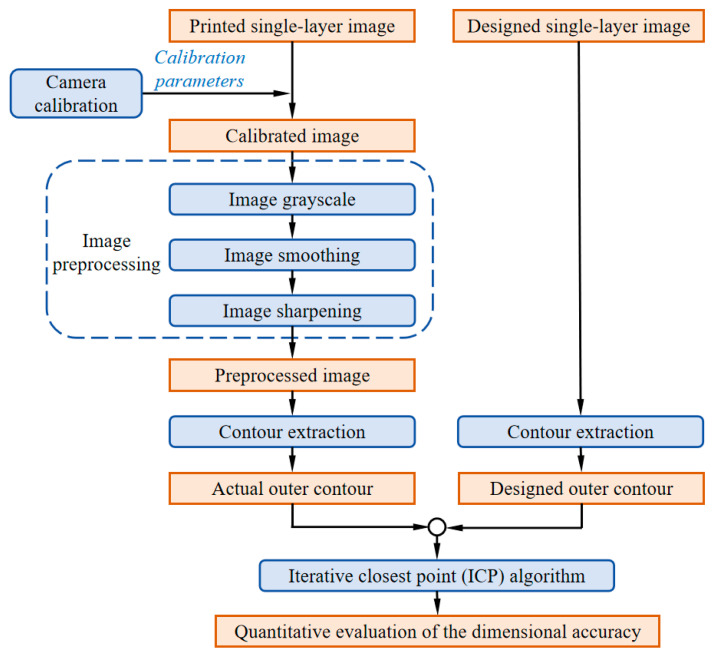
Designed image processing algorithm flow.

On the basis of the constructed hardware, the dimensional accuracy evaluation results of the printed single layer in DIW were obtained through image processing algorithms. As shown in Figure 4, the process steps of the image processing algorithm for evaluating the dimensional accuracy of the single layer in DIW based on machine vision were as follows.

(1) Obtain camera calibration parameters, including internal parameter matrix, external parameter matrix, and distortion parameters, through camera calibration;
(2) Generate the calibrated image by applying parameter corrections to the fabricated single-layer printed pattern;
(3) After calibration, the image is sequentially processed through image grayscale, image smoothing, and image sharpening to obtain the preprocessed image;
(4) Obtain the actual outer contour of the printed single layer in the preprocessed image through contour extraction algorithm;
(5) Obtain the outer contour of the printed single-layer design in the design image through contour extraction algorithm;
(6) Compare the actual outer contour with the designed outer contour and obtain a quantitative evaluation of the dimensional accuracy of the printed single layer through ICP algorithm.

### 3.3. Camera Calibration

The models that need to be calibrated for the camera included calibration model and distortion model. The calibration model was as follows [32]:

(2) $$Z_{c}\left[\begin{array}{c} x_{p}\\ y_{p}\\ 1 \end{array}\right]=\left[\begin{array}{c} \begin{array}{cccc} S_{x} & 0 & x_{0} & 0\\ 0 & S_{y} & y_{0} & 0\\ 0 & 0 & 1 & 0 \end{array} \end{array}\right]\left[\begin{array}{cc} R & T\\ 0_{1\times 3} & 1 \end{array}\right]\left[\begin{array}{c} X_{w}\\ Y_{w}\\ Z_{w}\\ 1 \end{array}\right],$$

where $Z_{c}$ is scale factor, $\left(X_{w},Y_{w},Z_{w}\right)$ is the coordinate of point P in the world coordinate system; $\left(x_{p},y_{p}\right)$ is the coordinate of point p in the image pixel coordinate system, which is converted by the camera imaging of point P; $S_{x}$ is the product of the number of pixels corresponding to the unit physical length in the horizontal direction of the image and the focal length; $S_{y}$ is the product of the number of pixels corresponding to the unit physical length in the vertical direction and the focal length; $x_{0}$ and $y_{0}$ are the horizontal and vertical coordinates of the origin of the image physical coordinate system in the image pixel coordinate system, respectively; R is the rotation matrix; T is the translation matrix; and $0_{1\times 3}$ is a zero matrix with one row and three columns.

The distortion models included the radial distortion model and the tangential distortion model, which were determined by Equations (3) and (4), respectively [33].

(3) $$\left\{\begin{array}{c} {\hat{x}}_{p}=x_{p}\left(1+k_{1}r^{2}+k_{2}r^{4}+k_{3}r^{6}\right)\\ {\hat{y}}_{p}=y_{p}\left(1+k_{1}r^{2}+k_{2}r^{4}+k_{3}r^{6}\right) \end{array}\right.,$$

(4) $$\left\{\begin{array}{c} {\hat{x}}_{p}=x_{p}+\left(2p_{1}y+p_{2}\left(r^{2}+2x^{2}\right)\right)\\ {\hat{y}}_{p}=y_{p}+\left(p_{1}\left(r^{2}+2y^{2}\right)+2p_{2}x\right) \end{array}\right.,$$

where $\left({\hat{x}}_{p},{\hat{y}}_{p}\right)$ is the pixel coordinate $\left(x_{p},y_{p}\right)$ of point P in the image pixel coordinate system after distortion compensation; r is the distance from the pixel point to the center point of the image, which can be expressed as $r=\sqrt{x_{p}^{2}+y_{p}^{2}}$. $k_{1}$, $k_{2}$ and $k_{3}$ are radial distortion parameters; and $p_{1}$ and $p_{2}$ are tangential distortion parameters.

The purpose of camera calibration was to obtain parameters $S_{x}$,$S_{y}$, $x_{0}$, $y_{0}$, $k_{1}$, $k_{2}$, $k_{3}$, $p_{1}$, and $p_{2}$.

Zhang’s calibration method was used to determine the parameters in the calibration model and distortion model. The calibration method constructs the relationship between the image pixel coordinates of the key points on the calibration board and the coordinates in the world coordinate system based on the pre-known key points on the calibration board and determines the calibration parameters based on the constructed camera model [34]. The specific steps were as follows.

(1) Prepare a calibration board with known key point positions and dimensions, change the camera’s position and angle relative to the calibration board, and obtain multiple calibration board images;
(2) Detect the key points in the calibration board image, obtain the pixel coordinates of the key points, and obtain the physical coordinate values of the key points on the calibration board based on the known positions and sizes of the key points on the calibration board;
(3) Assuming there is no distortion in the camera lens, the calibration model determined by Equation (2) is used to obtain the parameters $S_{x}$,$S_{y}$, $x_{0}$, and $y_{0}$;
(4) After determining the parameters in the calibration model, ignore the tangential distortion that has little impact and use the least squares method to obtain the parameters $k_{1}$ and $k_{2}$;
(5) Optimize the estimation using the maximum likelihood method to obtain the optimized calibration model parameters $S_{x}$,$S_{y}$, $x_{0}$, and $y_{0}$, as well as the radial distortion parameters $k_{1}$ and $k_{2}$.

**Figure 5:**
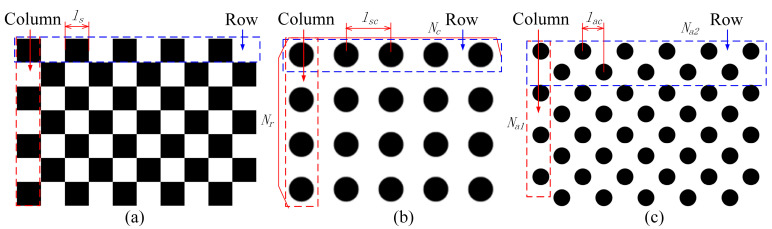
Common types of camera calibration boards and their key dimensions schematic diagram: (**a**) chessboard calibration board; (**b**) symmetric circle calibration plate; and (**c**) asymmetric circle calibration plate.

There are three commonly used calibration boards, as shown in Figure 5, which, respectively, illustrate the chessboard calibration board, symmetrical circle calibration board, and asymmetrical circle calibration board and their respective key dimensions. The chessboard calibration board consists of black and white symmetrically arranged squares, with the key dimension being the length of the squares, $l_{s}$. The symmetrical circle calibration board consists of symmetrically arranged black circles, with key dimensions of number of rows $N_{r}$, number of columns $N_{c}$, and circle spacing $l_{sc}$. The asymmetric circle calibration board consists of black circles arranged asymmetrically, with key dimensions including the number of circles $N_{a1}$ in the red dashed box, the number of circles $N_{a2}$ in the blue dashed box, and the circle spacing $l_{ac}$.

To select the optimal calibration board, three types of calibration boards were used for calibration. The key dimensions of the selected three calibration boards were the key dimensions of the chessboard calibration board were $l_{s}$ = 24 mm. The key dimensions of the symmetrical circle calibration plate were $N_{r}$ = 4 mm, $N_{c}$ = 5 mm, and $l_{sc}$ = 51 mm. The key dimensions of the asymmetric circle calibration plate were $N_{a1}$ = 4 mm, $N_{a2}$ = 11 mm, and $l_{ac}$ = 18 mm.

### 3.4. Image Preprocessing and Contour Extraction

Due to the inevitable interference of environmental lighting and detection electronic device noise, among other factors, during the acquisition, transmission, and storage of images, irrelevant noise information is mixed into the images, which reduces the image quality and ultimately leads to a decrease in the accuracy of single-layer contour extraction and dimensional accuracy evaluation results. Therefore, before extracting the contour of a single layer and evaluating its dimensional accuracy, it is necessary to perform image preprocessing operations, such as grayscale, smoothing, and sharpening, in order to improve image quality and ensure the accuracy of extracting the contour of a single layer. In this study, the maximum value method, average value method, and weighted average method were used to perform grayscale on color images to select the optimal grayscale method. Gaussian filtering, median filtering, and average filtering were, respectively, selected to perform image smoothing on grayscale images in order to select the optimal image smoothing method. Roberts operator, Sobel operator, Prewitt operator, and Laplacian operator were, respectively, selected to perform image sharpening on the smoothed grayscale image in order to select the optimal image sharpening method.

Contour extraction is used to obtain the outer contour of printed single-layer graphics, which is a prerequisite for evaluating the dimensional accuracy of the printed single layer. The principle of contour extraction method is to use the derivative of digital image to obtain the grayscale value changes between various pixel domains in the image and identify the pixels with rapidly changing grayscale values as contour edge points. This study used Roberts operator, Sobel operator, Prewitt operator, Laplacian operator, Laplace of Gaussian (LOG) operator, and Canny operator to extract the contour of printed single-layer graphics after image preprocessing in order to select the optimal contour extraction method.

To obtain the dimensional accuracy of a single layer, it is necessary to compare the actual outer contour with the designed outer contour in the physical coordinate system of the image. Due to the absence of lens distortion, noise interference, and other error factors for designing images in design software, it was already an ideal shape, the contour extraction method based on Canny operator was directly used to extract the outer contour of the single-layer design image.

### 3.5. Quantitative Acquisition of Single-Layer Dimensional Accuracy

In order to obtain quantitative dimensional accuracy of a printed single layer, this study proposes an ICP algorithm based quantitative dimensional accuracy evaluation method. The idea is to use the ICP algorithm to continuously approximate the actual outer contour and the designed outer contour through rigid body motion-based iterations, and the approximation error between the two contours is the dimensional accuracy of the printed single layer.

ICP algorithm is a registration method that aligns the actual measured contour with the design contour (standard contour) by iteratively optimizing rotation and translation parameters. The core lies in gradually reducing the spatial deviation between the two through multiple iterations, ultimately achieving high-precision matching.

The ICP algorithm is a point cloud-matching algorithm that minimizes the distance between two point sets through rotation and translation in order to achieve the goal of matching two point sets. During each iteration, the average sum of squares distance between all minimum distance points between two point sets can be obtained. Therefore, the point sets of the actual outer contour and the designed outer contour in the physical coordinate system of the image were used as the two point sets that needed to be matched. The two point sets were matched based on the ICP algorithm, and the average sum of squares of all the minimum distance points between the two point sets under the optimal iteration result was used as the quantitative dimensional accuracy evaluation result for the printed single layer. The specific process was as follows. See Algorithm 1.
**Algorithm 1:** Iterative closest point (ICP) algorithm.1: Define the point sets of the actual outer contour and the designed outer contour in the image physical coordinate system.2: Define the rotation matrix and translation vector for the iterative process.3: Define the objective function for each iteration.4: Define the iteration stopping condition.5: **If** objective function value < iteration stopping condition, **Then**
           **Stop,** dimensional accuracy = objective function value     **Else**
           **Repeat** to Step 3     **End**

Step 1: Define the point sets of the actual outer contour and the designed outer contour in the image physical coordinate system as $P_{C}$ = $\left\{p_{Ci}\right\}$ and $Q_{C}$ = $\left\{q_{Ci}\right\}$, respectively. $p_{Ci}$ and $q_{Ci}$ are the coordinate values of the points of the actual outer contour and the designed outer contour in the image physical coordinate system.

Step 2: Define the rotation matrix $R_{C}$ and translation vector $t_{C}$ for the iterative process. For the matching of the two-dimensional plane point set, $R_{C}$ and $t_{C}$ are represented by Equations (5) and (6), respectively.

(5) $$R_{C}=\left[\begin{array}{cc} \cos\theta_{Cx} & -\sin\theta_{Cx}\\ \sin\theta_{Cx} & \cos\theta_{Cx} \end{array}\right],$$

(6) $$t_{C}={\left[\begin{array}{cc} t_{Cx} & t_{Cy} \end{array}\right]}^{T},$$

where $\theta_{Cx}$ is the rotation angle of the planar shape relative to the horizontal axis and $t_{Cx}$ and $t_{Cy}$ are the translation components of the planar shape on the horizontal and vertical axes, respectively.

Step 3: Define the objective function for each iteration as Equation (7) and use singular value decomposition (SVD) method to obtain the optimal $R_{C}$ and $t_{C}$ for each iteration.

(7) $$\arg\underset{R_{C},t_{C}}{\min}\frac{1}{2}\sum_{i=1}^{N_{p}}{\left\|q_{Ci}-R_{C}p_{Ci}-t_{C}\right\|}^{2},$$

where $N_{p}$ is the number of points in the point set.

Step 4: Define the iteration stopping condition. If the iteration process meets the stopping condition, output the objective function value for the last iteration as dimensional accuracy. If the iteration process does not meet the condition, repeat Step 3 until the stopping condition is met.

In Step 4, the stopping condition indicator for iteration is the absolute value of the difference between the objective function values of the current iteration and the previous iteration. A basic rule of thumb is that measuring instruments should be at least ten times better than the process specifications they are measuring. Due to the repeatability accuracy of DIW is 0.05 mm, in order to ensure that the accuracy of single-layer dimensional accuracy evaluation results is greater than the repeatability accuracy of DIW, the threshold for stopping iterations needs to be set to be one order of magnitude higher than the repeatability accuracy. In this study, the iteration stopping condition for was set to the absolute value of the difference between the objective function values of the current iteration and the previous iteration being less than 0.001 mm.

## 4. Results and Discussion

### 4.1. Calibrated Camera Parameters

**Figure 6:**
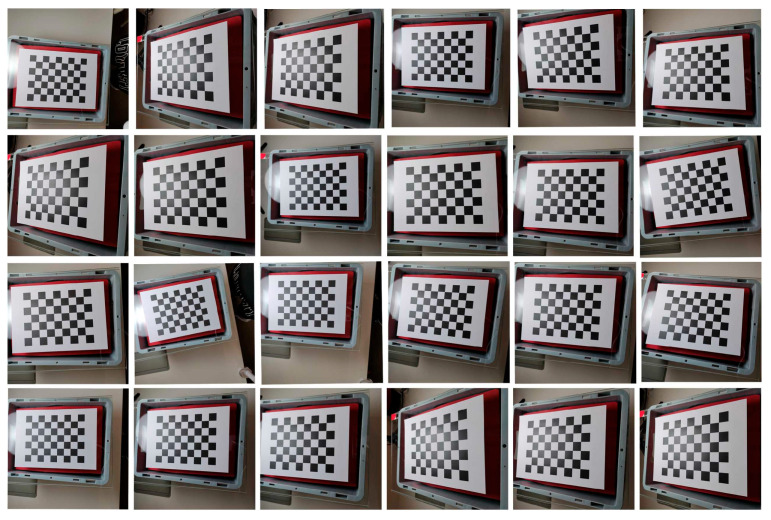
A total of 24 calibration images obtained based on the chessboard calibration board.

**Figure 7:**
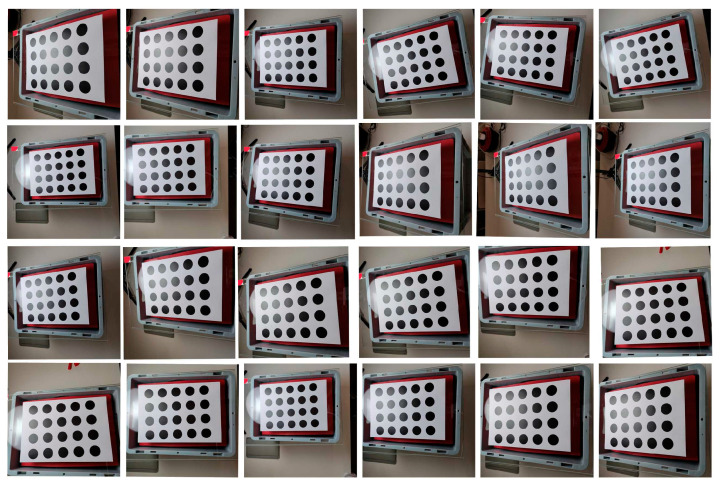
A total of 24 calibration images obtained based on a symmetrical circle calibration plate.

**Figure 8:**
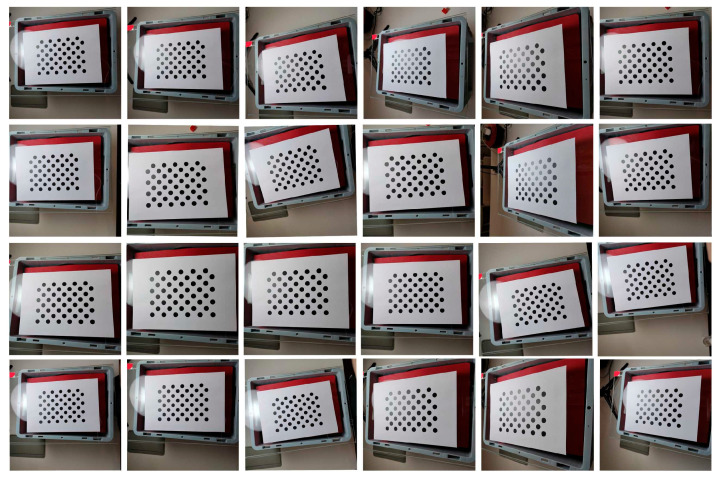
A total of 24 calibration images obtained based on asymmetric circle calibration plate.

According to the mathematical model of Zhang Zhengyou’s calibration method, in order to fully solve the internal parameters of the camera, at least three calibration plate images with different poses are required to meet the constraints of solving the linear equation system [35]. In practical applications, it is recommended to take 10–30 photos of the calibration plate at different angles and positions in order to improve calibration accuracy and optimize distortion parameters. To achieve comprehensive accuracy and efficiency, this article chooses to take 24 photos of the calibration board at different angles and positions [36]. As shown in Figure 6, Figure 7 and Figure 8, the position and relative camera posture of the three calibration plates were changed and 24 pictures for each calibration plate were taken. The calibration parameters were obtained based on the 24 images and listed in Table 2. The average pixel errors corresponding to the three calibration plates are listed in Table 3. It can be seen that the asymmetric circle calibration plate corresponded to the smallest average pixel error of 0.72 pixels, while the symmetric circle calibration plate corresponded to the largest average pixel error of 1.54 pixels. Therefore, the asymmetric circle calibration plate was selected as the calibration plate used in the camera calibration process in this study.

Therefore, based on the asymmetric circle calibration plate, the optimal camera calibration parameters obtained were $S_{x}$ = 3488.3 pixels, $S_{y}$ = 3485.6 pixels, $x_{0}$ = 1758.2 pixels, $y_{0}$ = 1757.0 pixels, $k_{1}$ = 0.0882, and $k_{2}$ = −0.2265.

### 4.2. Preprocessed Images and Extracted Contour

**Figure 9:**
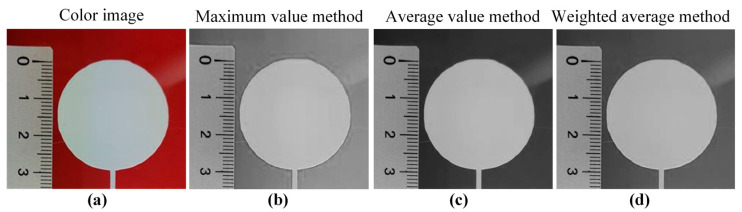
Grayscale images obtained based on three grayscale methods: (**a**) color image; (**b**) maximum value method; (**c**) average value method; and (**d**) weighted average method.

This study used a solid circle with a diameter of 30 mm as the shape for printed single layer and conducted experimental verification of the dimensional accuracy evaluation of single layers in DIW based on machine vision. Figure 9 showed the grayscale images obtained using three grayscale methods. The grayscale image obtained by the average method had the best contrast. Therefore, the average method was used to grayscale the color image.

**Figure 10:**
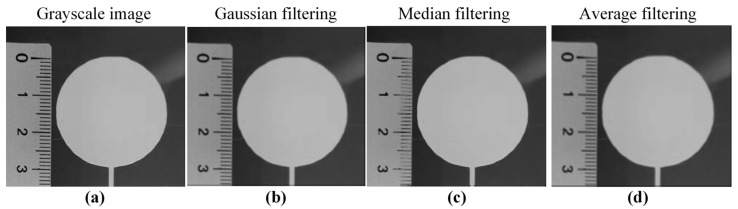
Smoothed grayscale images obtained based on three smoothing methods: (**a**) grayscale image before filtering; (**b**) Gaussian filtering; (**c**) median filtering; and (**d**) average filtering.

Figure 10 shows the comparison between the smoothed grayscale images obtained by three image smoothing methods. Median filtering made the contour edges the clearest. Therefore, median filtering was selected for image smoothing of the grayscale images.

**Figure 11:**
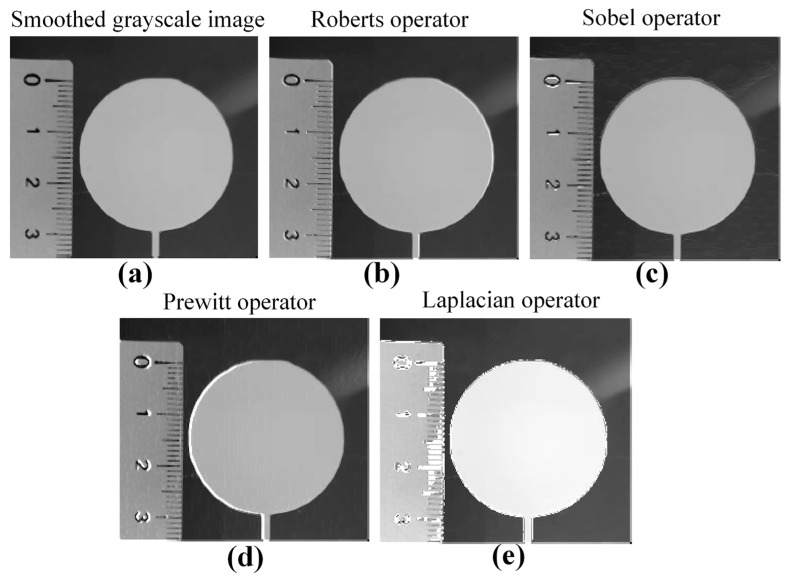
Sharpened smoothed grayscale images obtained based on four sharpened methods: (**a**) Smoothed grayscale image; (**b**) Roberts operator; (**c**) Sobel operator; (**d**) Prewitt operator; and (**e**) Laplacian operator.

Figure 11 showed the comparison of the sharpened images obtained by four image sharpening methods. The edge detection image sharpening method based on Laplacian operator made the contour edges the clearest. Therefore, the Laplacian operator-based edge detection image sharpening method was selected to perform image sharpening on the smoothed grayscale image.

**Figure 12:**
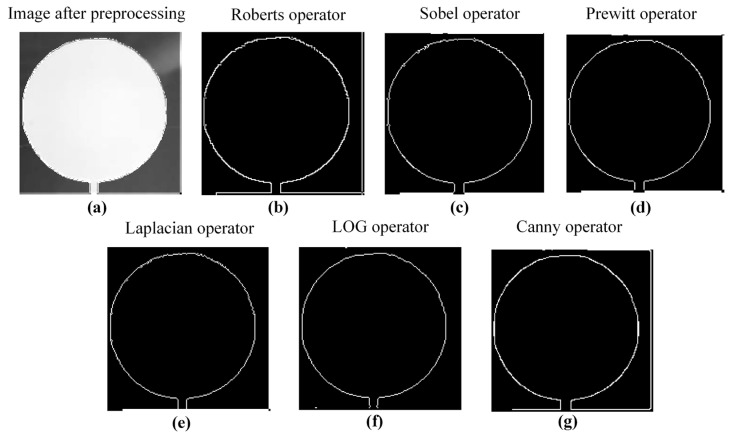
Contour extraction based on edge detection for printed single layers: (**a**) image after preprocessing; (**b**) Roberts operator; (**c**) Sobel operator; (**d**) Prewitt operator; (**e**) Laplacian operator; (**f**) LOG operator; and (**g**) Canny operator.

Figure 12 showed the contour extraction of printed single layer using Roberts operator, Sobel operator, Prewitt operator, Laplacian operator, LOG operator, and Canny operator, respectively. From the comparison of contour edges obtained by various edge detection methods in Figure 12 based on visual comparison, the contour edge of the printed single layer obtained based on the Canny operator was the clearest and most complete considering accuracy, detail preservation, and contour integrity comprehensively. Therefore, the Canny operator was selected to extract the contour of the printed single layer after image preprocessing.

### 4.3. Quantitative Dimensional Accuracy

**Figure 13:**
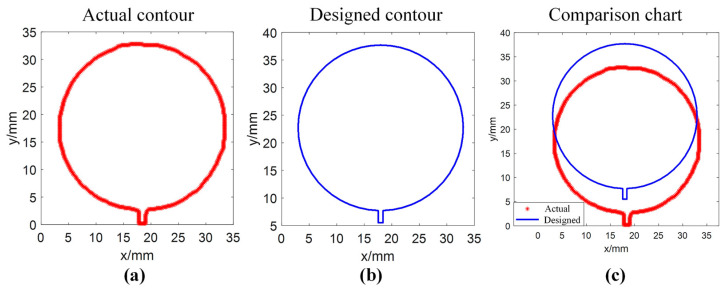
Comparison between the actual outer contour and the designed outer contour of the printed single layer in the physical coordinate system: (**a**) actual outer contour; (**b**) designed outer contour; and (**c**) comparison chart.

Figure 13a shows the actual contour of the printed single layer in the physical coordinate system. Figure 13b showed the designed contour of the single layer in the physical coordinate system. The actual contour and the designed contour were put directly into one image, and a comparison chart of the actual contour and designed contour was obtained as shown in Figure 13c.

**Figure 14:**
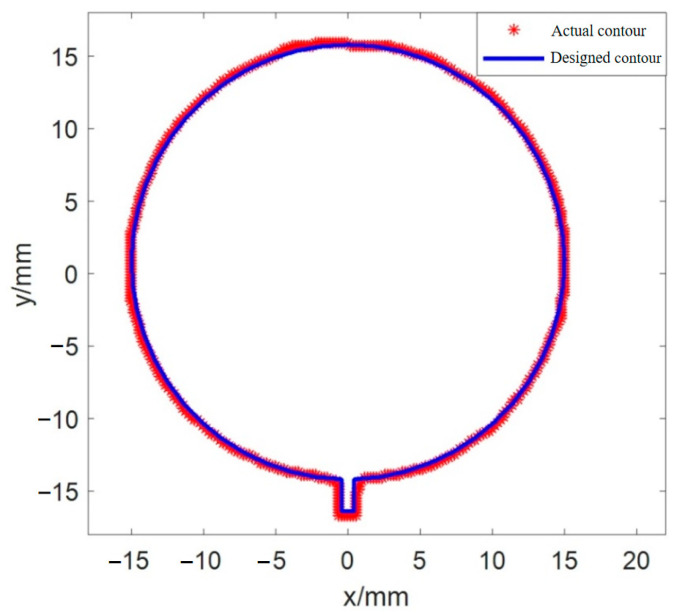
Matching result between the actual contour and designed contour of the printed single layer in the physical coordinate system.

Obviously, it was not possible to obtain quantitative dimensional accuracy of the printed single layer by comparing the actual contour and designed contour shown in Figure 13c. The ICP algorithm was used to obtain the matching result between the actual contour and designed contour, as shown in Figure 14, and taking the objective function value of the last iteration as the size accuracy of the printed single layer, the dimensional accuracy of the printed graphic corresponding to Figure 14 is obtained as 0.022 mm.

The accuracy comparison of the proposed method with previous studies is listed in Table 4. The accuracy of the proposed method was 0.001 mm, which is 80% higher than in previous methods. Thus, the proposed method based on image preprocessing and ICP algorithm can give a high accuracy dimensional accuracy evaluation for single layer in DIW.

### 4.4. Application in Comparison of Process Parameters

One of the applications of the dimensional accuracy evaluation method for printed single layer proposed in this study is to compare process parameters.

**Figure 15:**
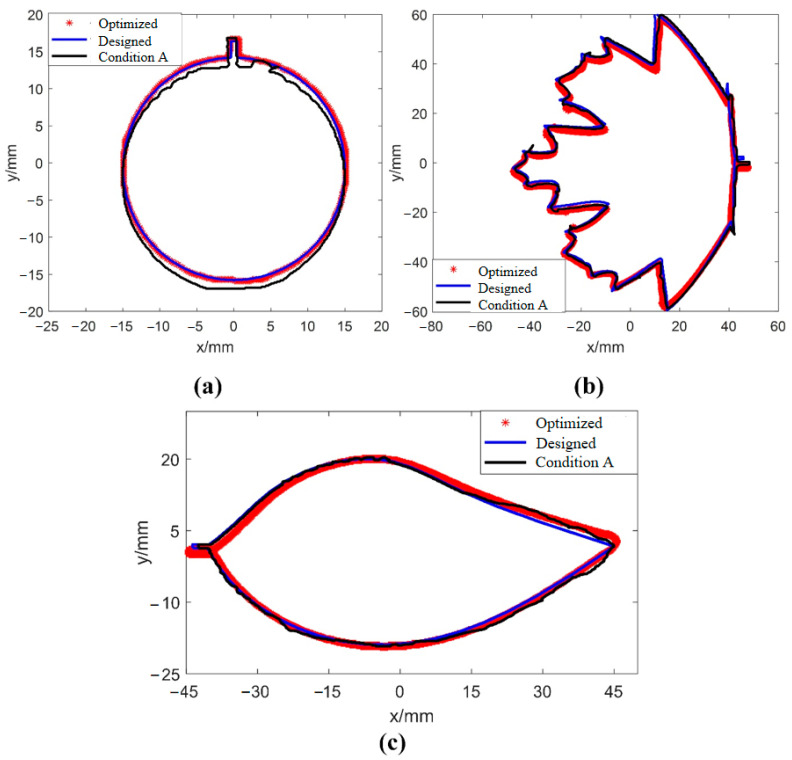
Matching result between the actual contour and designed contour of the printed single layer: (**a**) shape A; (**b**) shape B; and (**c**) shape C.

Three types of solid planar shapes, namely shape A (circular), shape B (leaf), and shape C (maple leaf), were printed using the optimized process parameters obtained in the literature [37] and the process parameters of condition A using the same ink and DIW printer in the literature [37]. A quantitative dimensional accuracy evaluation was conducted on the printed single layers under two different process parameter settings. The matching results between the actual contour and designed contour of printed single layers were shown in Figure 15, and the quantitative dimensional accuracy evaluation results of single layers were listed in Table 5. From Table 5, it can be seen that the dimensional accuracy of the printed single layer was improved by at least 76.3% using the optimized process parameters.

## 5. Conclusions

This study has conducted research on machine vision-based dimensional accuracy evaluation of single layer in DIW. The machine vision-based dimensional accuracy evaluation method proposed in this study can accurately and non-contact provide the dimensional accuracy of printed single-layer dimensional accuracy in DIW. The main findings are summarized as follows.

(1) In the camera calibration, the asymmetric circle calibration plate is selected as the calibration plate because the asymmetric circle calibration plate corresponded to the smallest average pixel error of 0.72 pixels, while the symmetric circle calibration plate corresponded to the largest average pixel error of 1.54 pixels.
(2) In the image preprocessing, the average method is used to grayscale the color image. The median filtering is selected for image smoothing of the grayscale images. The Laplacian operator-based edge detection image sharpening method is selected to perform image sharpening on the smoothed grayscale image.
(3) After image preprocessing, the Canny operator is selected to extract the contour of the printed single layer after image preprocessing considering accuracy, detail preservation, and contour integrity comprehensively.
(4) ICP algorithm is used to obtain quantitative dimensional accuracy evaluation results based on the processed and extracted contour. The accuracy of the proposed method for dimensional accuracy evaluation of the printed single layer in DIW is 0.001 mm, which is 80% higher than previous methods. Process parameter optimization experiments verifies the proposed method as the dimensional accuracy of printed single layers is improved by at least 76.3%.

Future work will be based on the method proposed in this article to obtain real-time printing accuracy and will use real-time accuracy for quality monitoring of printing processes based on deep learning for DIW. At the same time, future work will investigate multi-vision and obtain the accuracy evaluation results of 3D dimensions for DIW printed parts.

## Figures and Tables

**Table 1:** Summary of related works on machine vision based dimensional accuracy evaluation of different product types.

Study	Product Type	Technique Used	Result (Accuracy)
Sun et al. [26]	Shaft diameter	Machine vision method by improving the calibration model	0.005 mm
Li et al. [27]	Bottom radius and height of cylinders	A CCD camera system with preprocessing operations	0.01 mm
Dang et al. [28]	Large-size ring-shaped objects	Single-camera system by capturing objects with multiple partial images	0.063 mm
Tan et al. [29]	Shaft diameter	Structured light system composed of a laser linear light source and a camera	0.019 mm
Nogueira et al. [30]	Specifications on external contours	Monocular machine vision system by image processing	0.008 mm

**Table 2:** Calibration results obtained based on three calibration boards.

Calibration Parameters	Value		
	Chessboard Calibration Board	Symmetrical Circle Calibration Plate	Asymmetric Circle Calibration Plat
$S_{x}$/pixels	3498.7	3478.6	3488.3
$S_{y}$/pixels	3497.0	3490.2	3485.6
$x_{0}$/pixels	1757.4	1747.7	1758.2
$y_{0}$/pixels	1746.7	1773.6	1757.0
$k_{1}$	0.0303	0.0685	0.0882
$k_{2}$	−0.1624	−0.0478	−0.2265

**Table 3:** Average pixel error of calibration results obtained based on three calibration boards.

Calibration Board	Chessboard Calibration Board	Symmetrical Circle Calibration Plate	Asymmetric Circle Calibration Plat
Average pixel error/pixels	0.79	1.54	0.72

**Table 4:** Accuracy comparison of the proposed method with previous studies.

Study	Accuracy	Improvement
This study	0.001 mm	—
Sun et al. [26]	0.005 mm	80%
Li et al. [27]	0.01 mm	90%
Dang et al. [28]	0.063 mm	98%
Tan et al. [29]	0.019 mm	95%
Nogueira et al. [30]	0.008 mm	88%

**Table 5:** Comparison results of quantitative dimensional accuracy of a single layer under condition A and optimized parameters.

Shape	Dimensional Accuracy/mm		Precision Improvement/%
	Condition A	Optimized Parameters	
Shape A	0.63	0.02	96.8
Shape B	0.38	0.09	76.3
Shape C	0.89	0.12	86.5

## Data Availability

The data will be made available upon request.

## References

[1] Bigliardi B., Bottani E., Gianatti E., Monferdini L., Pini B., Petroni A. (2024). Sustainable Additive Manufacturing in the context of Industry 4.0: A literature review. Procedia Comput. Sci..

[2] Gao G., Wang Y., Wang Y., Zhang K., Xiang D., Ma J. (2025). Development on shape and performance control of aeronautical parts in additive manufacturing. J. Manuf. Process..

[3] Tu Y., Zhang H., Shi X., Fan J., Bao B., Lu G., Han F., Wu H., Hassan A. (2025). Numerical Prediction and Experimental Validation of Deposited Filaments in Direct Ink Writing: Deposition Status and Profile Dimension. Polymers.

[4] Shen A., Caldwell D., Ma A.W.K., Dardona S. (2018). Direct write fabrication of high-density parallel silver interconnects. Addit. Manuf..

[5] Guzi de Moraes E., Ferreira I.M., Teixeira L.B., Cartapati L.H., Souza M.T., Novaes de Oliveira A.P. (2022). Additive manufacturing of cellular structures from recycled soda-lime glass printing inks by robocasting. Ceram. Int..

[6] Cesarano J., Calvert P. (2000). Freeforming Objects with Low Binder Slurry. U.S. Patent.

[7] Ang X., Tey J.Y., Yeo W.H., Shak K.P.Y. (2023). A review on metallic and ceramic material extrusion method: Materials, rheology, and printing parameters. J. Manuf. Process..

[8] Feng H., Wang S., Cui Y., Xiang Y., Liu X., Sun X., Zhang W., Tu P. (2023). Effect of 3D printing process parameters on the mechanical properties of silica/polyethyleneimine composites using direct-ink writing. Polym. Compos..

[9] Sun L., Parker S.T., Syoji D., Wang X., Lewis J.A., Kaplan D.L. (2012). Direct-write assembly of 3D silk/hydroxyapatite scaffolds for bone co-cultures. Adv. Healthc. Mater..

[10] Sun K., Wei T.-S., Ahn B.Y., Seo J.Y., Dillon S.J., Lewis J.A. (2013). 3D Printing of Interdigitated Li-Ion Microbattery Architectures. Adv. Mater..

[11] Lessing J., Glavan A.C., Walker S.B., Keplinger C., Lewis J.A., Whitesides G.M. (2014). Inkjet Printing of Conductive Inks with High Lateral Resolution on Omniphobic “RF Paper” for Paper-Based Electronics and MEMS. Adv. Mater..

[12] Frutiger A., Muth J.T., Vogt D.M., Mengüç Y., Campo A., Valentine A.D., Walsh C.J., Lewis J.A. (2015). Capacitive Soft Strain Sensors via Multicore–Shell Fiber Printing. Adv. Mater..

[13] Sydney Gladman A., Matsumoto E.A., Nuzzo R.G., Mahadevan L., Lewis J.A. (2016). Biomimetic 4D printing. Nat. Mater..

[14] Wehner M., Truby R.L., Fitzgerald D.J., Mosadegh B., Whitesides G.M., Lewis J.A., Wood R.J. (2016). An integrated design and fabrication strategy for entirely soft, autonomous robots. Nature.

[15] Zhou N., Liu C., Lewis J.A., Ham D. (2017). Gigahertz Electromagnetic Structures via Direct Ink Writing for Radio-Frequency Oscillator and Transmitter Applications. Adv. Mater..

[16] Blanco-Angulo C., Martínez-Lozano A., Arias-Rodríguez J., Rodríguez-Martínez A., Vicente-Samper J.M., Sabater-Navarro J.M., Ávila-Navarro E. (2023). Low-Cost Direct-Writing of Silver-Based Ink for Planar Microwave Circuits up to 10 GHz. IEEE Access.

[17] Jiang T., Lin Z., Qiao X., Yang Y., Hong Y., Shang J., Luo Z., Matthew Kinsella J. (2023). A dual-index quality evaluation method for direct ink writing of soft materials. Mater. Lett..

[18] Belgin Paul D.L., Praveen A.S., Arjunan A. (2025). Parametric optimisation for 3D printing β-tricalcium phosphate tissue engineering scaffolds using direct ink writing. Smart Mater. Manuf..

[19] Guida L., Romani A., Negri D., Cavallaro M., Levi M. (2025). 3D-printable PVA-based inks filled with leather particle scraps for UV-assisted direct ink writing: Characterization and printability. Sustain. Mater. Technol..

[20] Legett S.A., Torres X., Schmalzer A.M., Pacheco A., Stockdale J.R., Talley S., Robison T., Labouriau A. (2022). Balancing Functionality and Printability: High-Loading Polymer Resins for Direct Ink Writing. Polymers.

[21] Belyaeva A.A., Eksakusto P.O., Morozova S.M. (2024). Thermally and magnetically responsive single layer bioinspired soft actuator with patterned structure obtained by direct ink writing. Mater. Today Commun..

[22] Xu J., Buswell R.A., Kinnell P., Biro I., Hodgson J., Konstantinidis N., Ding L. (2020). Inspecting manufacturing precision of 3D printed concrete parts based on geometric dimensioning and tolerancing. Autom. Constr..

[23] Wi K., Suresh V., Wang K., Li B., Qin H. (2020). Quantifying quality of 3D printed clay objects using a 3D structured light scanning system. Addit. Manuf..

[24] Petsiuk A.L., Pearce J.M. (2020). Open source computer vision-based layer-wise 3D printing analysis. Addit. Manuf..

[25] Shen H., Sun W., Fu J. (2019). Multi-view online vision detection based on robot fused deposit modeling 3D printing technology. Rapid Prototyp. J..

[26] Sun Q., Hou Y., Tan Q., Li G. (2013). A Planar-Dimensions Machine Vision Measurement Method Based on Lens Distortion Correction. Sci. World J..

[27] Li B. (2018). Research on geometric dimension measurement system of shaft parts based on machine vision. Eurasip J. Image Video Process..

[28] Dang A.-T., Hsu Q.-C., Truong T.-T. (2021). A simple method for dimensional measurement of ring-shaped objects using image processing technique. Int. J. Adv. Manuf. Technol..

[29] Tan Q., Kou Y., Miao J., Liu S., Chai B. (2021). A Model of Diameter Measurement Based on the Machine Vision. Symmetry.

[30] Nogueira V.V.E., Barca L.F., Pimenta T.C. (2023). A Cost-Effective Method for Automatically Measuring Mechanical Parts Using Monocular Machine Vision. Sensors.

[31] Liu Y., Lv Z., Zhang Q., Zhao J., Fang Z., Gao Z., Su Y. (2023). Comparison Study of Three Camera Calibration Methods Considering the Calibration Board Quality and 3D Measurement Accuracy. Exp. Mech..

[32] Huang J., Liu S., Liu J., Jian Z. (2024). Camera calibration optimization algorithm that uses a step function. Opt. Express.

[33] Yang L., Zhou F., Zhang W., Liu Y. (2023). A novel camera calibration method based on circle projection model. Measurement.

[34] Zhang Z. (2000). A flexible new technique for camera calibration. IEEE Trans. Pattern Anal. Mach. Intell..

[35] Zhao Z., Zhu Y., Li Y., Qiu Z., Luo Y., Xie C., Zhang Z. (2020). Multi-Camera-Based Universal Measurement Method for 6-DOF of Rigid Bodies in World Coordinate System. Sensors.

[36] Liu X., Tian J., Kuang H., Ma X. (2022). A Stereo Calibration Method of Multi-Camera Based on Circular Calibration Board. Electronics.

[37] Tu Y., Arrieta-Escobar J.A., Hassan A., uz Zaman U.K., Siadat A., Yang G. (2021). Optimizing Process Parameters of Direct Ink Writing for Dimensional Accuracy of Printed Layers. 3D Print. Addit. Manuf..

